# Supplementary material for: Are carbon emissions trading and green financial instruments synergistic? -Comprehensive quantitative research based on content analysis
Source: PLoS One. 2024 Mar 7;19(3):e0298601. doi: 10.1371/journal.pone.0298601 (PMC10919601; doi:10.1371/journal.pone.0298601)
Supplement: S1 Data — (ZIP) [file pone.0298601.s001.zip › Data/地级市Z_F工作报告.zip!/╕╩╦α/╜≡▓2╩╨/└┤╫╘╣1⁄2╓┌║┼í╛┬φ┐╦╩2╛▌═°í┐_2011.docx]

各位代表：

　　现在，我代表市人民政府，向大会作工作报告，请予审议，并请政协委员和列席人员提出意见。

　　一、市六届政府工作回顾

　　过去的五年，是我市发展历程中不平凡的五年。面对不断变化的外部环境和纷繁复杂的改革建设任务，六届政府在省委、省政府和市委的坚强领导下，在市人大和市政协的监督支持下，深入贯彻落实科学发展观，聚精会神搞建设，一心一意谋发展，为实现科学发展、和谐发展、跨越发展奠定了坚实基础。

　　——经济持续快速增长，综合实力大幅提升。预计到2011年末，全市生产总值达到230亿元，是2006年152.6亿元的1.5倍，年均增长12.6％；人均生产总值7500美元，是2006年4113美元的1.8倍，年均增长11.9％；全社会固定资产投资130亿元，是2006年51.8亿元的2.5倍，年均增长20.2％；大口径财政收入27亿元，是2006年30.6亿元的88.2％，年均下降2.5％；地方财政收入11.4亿元，是2006年6.1亿元的1.9倍，年均增长13.2％；财政支出25.8亿元，是2006年11.5亿元的2.2倍，年均增长17.7％；社会消费品零售总额43亿元，是2006年20.6亿元的2.1倍，年均增长15.9％；城镇居民人均可支配收入19820元，是2006年11256元的1.76倍，年均增长12％；农民人均纯收入6670元，是2006年4293元的1.55倍，年均增长9.2 ％；进出口贸易总额45亿美元，是2006年17.7亿美元的2.5倍，占全省进出口贸易总额的50％，年均增长20.5％。金川集团公司位列2010年中国企业500强第92位、制造业500强第34位，八冶建设集团公司位列中国建筑业500强第381位，金化集团公司、瓮福化工公司、金铁集团公司、金泥集团公司和新川化工公司发展成为甘肃省100强企业。我市已成为省内最具生机活力和发展潜力的地区之一。

　　——结构调整成效明显，发展方式不断优化。工业主导地位更加牢固。2011年，规模以上工业增加值预计完成163亿元，是2006年115.5亿元的1.4倍，年均增长14％。镍、铜、钴和化工等工业主导产品产能大幅提升，分别达到15万吨、60万吨、1万吨和400万吨；循环经济发展亮点凸显，有色金属、化工等7条循环经济产业链不断延伸，形成了独具金昌特色的循环经济发展格局，我市被列为甘肃省循环经济示范区，金川集团公司被列为全国首批循环经济示范企业。金昌经济开发区升级为国家级经济技术开发区，金川新材料工业园、河西堡化工循环经济产业园和永昌工业园初具规模，形成了“一区三园”的发展格局。我市先后被确定为新材料产业国家高技术产业基地、国家新型工业化产业示范基地。节能减排成效明显，先后关停所有小火电机组、水泥立窑生产线和造纸厂，建成烟气治理、污水和垃圾处理等一批环保项目，提前一年完成“十一五”节能减排目标。农村经济稳步发展。2011年，全市农业增加值预计完成12.2亿元，是2006年7.86亿元的1.55倍，年均增长3.1％。五年公共财政涉农资金投入累计达到24.8亿元，年均增长31.4％。特色产业种植面积达到90％以上，优质肉羊、啤酒大麦、无公害蔬菜、食用菌等优质高效节水现代特色农业快速发展。培育农业产业化市级龙头企业30家、省级17家、国家级1家，70％以上的农产品实现加工增值，农业标准化覆盖率达到80％，农业机械化综合作业水平达到76％，高于全国和全省平均水平。第三产业快速发展。2011年，第三产业增加值预计达到36.8亿元，是2006年21.7亿元的1.7倍，年均增长9.8％。居民消费结构不断升级，以家用汽车、住房、通讯等为热点的消费升级趋势明显，特别是城市居民家庭百户拥有家用汽车由2006年的9辆增加到25辆，城镇人均住房面积由2006年的25平方米增加到30.5平方米。“家电下乡”活动拉动农村消费市场，汽车、电脑、空调等现代消费品走进寻常百姓家，农村消费潜力有效释放。地方饮食文化在传承中不断创新，成为绿色安全健康饮食消费主流。

　　——基础设施日益完善，城乡面貌显著改善。五年累计完成全社会固定资产投资462亿元。基础设施建设力度不断加大，金永高速公路建成通车，金昌机场顺利通航，金阿铁路一期工程全线贯通，金武高速公路开工建设，初步形成了公路、铁路、航空三位一体的立体化交通网络。城市服务功能进一步增强，新建改建市区城市道路99.16公里，城市环路和城区路网基本形成；先后建成市文化中心、游泳馆等一批公共服务设施和金水湖景区、龙泉景观带一期等一批城市景观工程，改造了人民文化广场、体育公园和金川公园，我市被命名为“全国无障碍设施建设先进城市”。城乡一体化走在全省前列，新建集中居住区56个、高标准农宅21718套，城镇化率由2006年的56.7％提高到62.1％；解决了10.5万人的饮水不安全问题，85％的农户用上了自来水；累计改造农村危旧房19015户，新建沼气池9501座，100％的行政村通了油路或水泥路，所有乡镇建成文化体育广场，所有乡镇和72％的村委会新建了办公场所。造林绿化工程深入实施，森林覆盖率达到20.82％，市区绿化覆盖率达到33.46 ％，人均公共绿地面积达到18.3平方米，我市被命名为甘肃园林城市。

　　——各项改革深入推进，发展活力不断增强。政府机构改革和乡镇机构改革全面完成，事业单位分类改革稳步推进。国有企业改革成效显著，资本运营和资产重组有了突破性进展，金川国际板块成功进入香港资本市场；引进新希望集团组建了甘肃新川化工公司，金化集团公司与贵州瓮福集团公司出资组建甘肃瓮福化工公司，并引进中化集团公司参股经营，实现了股权多元化；企业分离办社会工作加快推进，八冶公司、金化集团公司、金昌毛纺厂政策性破产工作基本完成。非公经济快速发展，在活跃地方经济、扩大就业渠道等方面发挥了重要作用。改革政府投资项目管理模式，政府投资项目推行代建制。人才工作体制机制不断创新，人才工作力度持续加大，人才资本快速汇集机制逐步形成。医药卫生体制改革稳步推进，公立医院改革全面启动，基层医疗卫生机构全面实行基本药物零差价制度，城乡居民住院医疗费用政策范围内报销比例达到70％以上。统筹城乡综合配套改革试点工作扎实推进，取消城乡户口类别，集体林权确权率达到100％，水权确权工作全面完成，农村土地流转率达到17％，金川区被确定为全国农村土地承包经营权登记试点区。《金昌—武威区域经济一体化发展规划》启动实施。对外开放步伐加快，与美国邵尼市等城市缔结了友好合作关系。

　　——民生保障力度加大，社会事业全面进步。五年来，民生事业累计投入39.6亿元，年均增长26.4％。千方百计扩大就业，在全省率先建立了城乡一体的人力资源和就业服务体系，应届高校毕业生年就业率达到85％以上，城镇登记失业率控制在4％以内。城乡社会保障水平居全省前列，城市低保标准提高到230元／月，农村低保标准达到1100元／年；在全省率先建立了城乡一体的社会救助体系，国有企业职工养老保险实现省级统筹，医疗保险、失业保险、工伤保险和生育保险实现市级统筹；率先实现城乡居民基本医疗保险一体化，参保率分别达到99.08％和95.08％，提前实现省上提出的“四个全覆盖”目标。新型农村社会养老保险参保率达到98.07％。大力推进科技进步，科技对经济增长的贡献率达到57％。城乡义务教育均衡发展，教育资源布局不断优化，校安工程加快实施，撤并农村中小学63所，建成寄宿制学校25所，新建城市学校9所，改扩建校舍面积25.89万平方米，消除危房25万平方米，在全省率先普及高中阶段教育，率先免除中等职业教育学费和城乡中小学学杂费；甘肃有色冶金职业技术学院一期工程顺利建成，实现首批招生目标，填补了金昌无高等院校的空白。医疗卫生服务体系建设不断加强，医疗卫生条件显著改善，金川公司职工医院门诊楼、八冶公司职工医院门诊楼、市疾控中心检验楼和市人民医院门诊楼、传染病区、精神病区相继建成投入使用，所有乡镇卫生院建起了业务楼，所有行政村和社区建有卫生室或卫生服务中心，村卫生室执业医师达到80％。大力弘扬“艰苦奋斗、和谐进取”的金昌精神，深入开展群众性精神文明创建活动，我市先后被命名为全国未成年人思想道德建设工作先进城市、省级文明城市标兵，第三次荣获“全国创建文明城市工作先进城市”称号。全面完成市区数字电视转换工作，农村广播、电视覆盖率分别达到85.8％和91.4％，所有行政村建有“农家书屋”和文体活动场所，中央电视台“激情广场•爱国歌曲大家唱”专题节目两次来我市演出。认真落实计划生育基本国策，75％的乡镇建成了高标准计生服务所，我市被评为“全国计划生育优质服务先进市”。

　　——政府自身建设加强，行政水平不断提高。自觉接受人大依法监督和政协民主监督，共办理人大代表意见建议、政协委员提案1435件，办复率100％。深入开展学习实践科学发展观和“创先争优”活动，扎实开展政风行风评议活动，机关作风进一步好转。积极推行政务公开和政府信息公开工作，修订完善政府工作规则和重大事项决策程序，建立了重大决策社会听证、社会公示和专家咨询等20多项制度，政府理政能力不断提高。行政审批制度改革取得新突破，共清理行政许可项目844项，取消285项，精简34％，行政审批行为不断规范。认真开展“小金库”、工程建设领域突出问题和党政机关公务用车专项治理工作，建立了政府采购、工程招投标、社保资金管理等5个电子监督平台。认真落实领导干部行政问责制，不断加强对重大决策、重点工作和关系民生问题的政务督查和执法监察，行政效能不断提高。积极推进平安金昌、法治金昌创建工作，法律服务、法律援助工作不断加强，“五五”普法任务全面完成，应急管理体系基本建立，信访案件到期办结率达91％，社会治安防控体系逐步完善，人民群众的安全感进一步增强。农业普查、经济普查和第六次人口普查任务圆满完成，二轮地方志编纂工作有序推进，人防、档案、侨务、审计、气象、新闻出版、国土资源、防震减灾、民族宗教、妇女儿童等各项事业都有了新的发展。

　　各位代表，2011年，我们认真贯彻落实省委、省政府和市委的决策部署，紧紧围绕市六届人大七次会议确定的目标任务，大力发展循环经济不动摇，扎实推进城乡一体化不松懈，坚持“五城联创”不停步，保障和改善民生不放松，全市经济社会平稳较快发展。预计生产总值同比增长15.5％，规模以上工业增加值增长17.5％，固定资产投资增长20％，大口径财政收入增长14.9 ％，地方财政收入增长11.5％，社会消费品零售总额增长17％，城镇居民人均可支配收入、农村居民人均纯收入分别增长12.1％和12％。

　　一是工业经济快速发展。优势支柱产业效益和质量稳步提升，预计规模以上工业企业销售收入、利润总额分别增长36％和40％，综合效益指数提高5个百分点。金川集团公司荣获“中国工业大奖”，成为中国有色金属行业和甘肃省唯一获此殊荣的企业。市财政安排专项资金支持市属企业上市，金泥集团公司等企业上市工作顺利推进。氟化工产业快速兴起，产能达到6万吨。新能源产业加快发展，三峡新能源公司一期10万千瓦风电、中科5兆瓦光伏发电项目开工建设。工业固废综合利用步伐加快，110万吨铜炉渣选矿项目顺利实施，我市被命名为全国工业固废综合利用示范基地。污染治理成效明显，永昌县污水处理工程投入试运行，市区环境空气质量持续好转，首次达到国家二级标准。

　　二是农业和农村经济稳步提升。编制完成《农业和农村区域“十二五”发展规划》。完成农作物播种面积106.5万亩，粮食总产量达到33.7万吨。安排专项资金支持“菜篮子”工程建设和草食畜牧业发展，蔬菜自给能力进一步提高，畜禽饲养总量预计达到230万头（只）。建立了水利建设基金筹措和使用管理机制，西金干渠改扩建、金川峡水库淹没区补偿与围栏保护等项目顺利实施。输转城乡富余劳动力8.2万人次，实现劳务收入8亿元，劳务输转已成为农民增收的重要渠道。积极推进以工促农、地企共建，各大企业帮扶新农村建设资金物资折合2500多万元。

　　三是重点项目建设顺利推进。实行重大项目市级领导包抓责任制、固定资产投资目标责任制和工作例会制，全市在建项目254个，其中新开工项目158个。金川集团公司20万吨铜、1.5万吨海绵钛、1万吨钴、5000吨羰基铁建成投产，鑫华焦化150万吨捣固焦及300万吨重介洗煤项目基本建成，市区2&times;330兆瓦热电联产、金川集团公司6万吨电解镍、金化集团公司20万吨合成氨、金泥集团公司40万吨电石、镕凯20万吨电石、嘉森60万吨洗煤等项目加快建设，市区天然气燃气工程建成投运，车用天然气利用工程启动实施。切实加大招商引资力度，引进项目92个，到位资金23亿元。

　　四是第三产业发展势头良好。编制完成《第三产业发展规划》，“万村千乡”市场工程深入实施，太西煤5000万吨物流园区、大润发超市、北京路市场和金三角市场改扩建等项目前期工作顺利推进。全市17个商标被评为“甘肃省著名商标”，8个产品获得“甘肃名牌产品”称号。加快发展旅游业，建立了旅游发展基金，骊靬古城建设进展顺利，成功举办了“丝绸之路•骊靬文化”国际旅游研讨会，骊靬文化引起国内外广泛关注。与张掖、武威、阿右旗等周边城市的旅游合作有了实质性进展，“农家乐”星级划分与标准评定通过省级评审。

　　五是城市建设步伐加快。深入开展“五城联创”，切实加强城市规划建设和管理，编制完成城乡总体规划和龙泉景观带三期、金永高速公路连接线绿化景观规划。西宁路等7条城区道路开工建设，市人力资源和社会保障服务大厅全面建成，金昌监狱、金昌汽车客运站、市残疾人综合服务中心、市儿童福利康复中心和市流浪儿童救助中心即将完工，开发区综合服务中心、金昌大剧院加快建设。制定出台公共场所禁止吸烟、禁止随地吐痰随地丢弃废弃物等管理制度，城市管理常态化、长效化机制不断完善。全国文明城市国家测评工作已经结束；国家卫生城市顺利通过综合评审和公示，即将正式命名；顺利通过第六次创建“全国双拥模范城”工作考核验收；我市被命名为“全国残疾人工作示范城市”，被评为“全国残疾人工作先进单位”。

　　六是民生事业不断进步。多渠道开发就业岗位，新增城镇就业8409人，零就业家庭动态消零。社会保险覆盖面继续扩大，城镇居民社会养老保险试点工作全面启动。学前教育三年行动计划启动实施，“两基”目标全面实现。全民健康工程、体育健身长廊工程有序推进，建立了文化事业和文化产业发展基金，我市被列为全国首批国家公共文化服务体系示范区创建城市和全省首批体育惠民工程示范市。成功举办了建市30周年系列庆典活动。市财政安排专项资金，建立了价格调节基金和低保标准与物价挂钩机制。保障性住房建设走在全省前列，省上下达的2540套保障性住房全部开工建设，主体竣工率达到80％。深入开展食品添加剂监管专项行动，启动实施“放心粮油”工程，建成全市首个禽类定点屠宰场。

　　各位代表，我们圆满完成了“十一五”规划的各项任务，实现了“十二五”发展的良好开局。过去五年发生的深刻变化和取得的突出成就，凝聚着全市人民的心血和汗水，凝聚着社会各界的智慧和力量。在此，我代表市六届人民政府，向在各行各业和各条战线上付出辛勤劳动、做出积极贡献的全市广大干部群众，向关心支持金昌现代化建设的各族各界人士，表示衷心的感谢和崇高的敬意！

　　各位代表，过去五年取得的成绩令人鼓舞，发展的形势催人奋进，但跨越发展、区域竞争、富民强市的压力依然很大，前进道路上还有不少矛盾和问题：一是水资源紧缺，农业基础薄弱，产业化程度低，农民持续增收难度增大。二是经济结构不尽合理，第三产业总量小、层次低、发展慢。三是工业单位生产总值能耗高，节能减排任务重，环境空气质量稳定达标难度大。四是固定资产投资总量小、大项目少、增速慢，对经济发展的拉动作用不够。五是居民消费价格指数比较高，平抑物价压力大。六是部分政府工作人员的责任意识、服务意识、工作效能还不适应发展的新要求，抓项目力度不够大，等等。我们必须直面人民群众的关切，直面各方面的监督，直面政府自身工作中的不足，勇于迎接挑战，敢于不断改进，善于主动应对，切实解决好存在的问题，绝不辜负人民的重托和期望。

　　二、今后五年政府工作的总体思路和奋斗目标

　　面向未来，金昌已经站在一个新的历史起点上。从所处阶段看，我市农业从传统农业向现代农业转变；工业从主要依靠资源开发向依靠科技进步转变；服务业从满足基本需求向高层次、多样化消费和生产性服务转变；社会保障从低水平、广覆盖向高水平、全覆盖转变；城乡经济社会发展从“二元结构”向城乡一体化转变。这种阶段性特征决定了今后五年是金昌全面建设小康社会、实现跨越发展的关键时期，也是加快转变发展方式、实现可持续发展的攻坚时期。从有利条件看，全球范围内的资本流动和产业转移，为我们更好地集聚外部资源创造了有利条件；国家支持甘肃发展的优惠政策、新一轮西部大开发、金昌列入全国老工业基地调整改造规划范围、省委省政府确定的区域发展战略，为我们跨越发展提供了难得的历史机遇；经过多年努力，我市产业基础更加牢固，比较优势更加明显，发展环境更加优化，为我们跨越发展奠定了坚实基础；建市30年积累的宝贵经验和过去五年发展的生动实践，进一步坚定了全市人民推动改革开放事业的信心；我市形成的风正气顺、人和业兴的良好氛围，为我们跨越发展提供了根本保证。我们必须认清形势、正视困难，抢抓机遇、锐意进取，努力推动经济社会跨越式科学发展。

　　市第七次党代会确定了我市今后五年工作的指导思想、奋斗目标和战略举措，这是做好政府工作的行动纲领。我们将以科学发展观为指导，按照省委、省政府“四抓三支撑”的总体思路和区域发展战略要求，认真贯彻落实市第七次党代会精神，紧紧围绕资源型城市可持续发展“一个主题”，突出循环经济和城乡一体化 “两个重点”，加快新型工业化、农业现代化和城镇化“三化进程”，一手抓跨越发展，一手抓保障民生，全力打造科学发展的先导区、统筹发展的先行区、和谐发展的示范区。

　　主要奋斗目标是：生产总值年均增长15％，规模以上工业增加值年均增长18％，全社会固定资产投资年均增长20％，大口径财政收入年均增长15％，地方财政收入年均增长15％，社会消费品零售总额年均增长18％，进出口贸易总额年均增长20％，城镇居民人均可支配收入年均增长14％，农民人均纯收入年均增长13％，居民消费价格总水平涨幅控制在4％左右。

　　为实现上述目标，今后五年政府工作要在经济社会发展的重点领域和关键环节上实现新跨越。

　　在加快新型工业化上实现新跨越。坚定不移地改造提升壮大传统优势产业，坚定不移地发展新兴产业，坚定不移地发展循环经济，注重存量升级、增量创新，促进工业结构优化升级，初步形成优势产业集群。力争到2016年实现“11521”目标，有色金属初级产品达到100万吨以上，各类化工产品达到1000万吨，有色金属加工材超过50万吨，光伏并网电站规模达到200兆瓦，风力发电规模达到1000兆瓦，把我市建成西北地区重要的基础化工基地、全国最大的镍钴铜有色金属粉体材料生产基地和有色金属新材料循环经济基地，使有色冶金新材料加工增加值超过现有有色金属原材料加工增加值，为全省到2015年建成全国循环经济发展示范区发挥率先带头作用。

　　在统筹城乡发展上实现新跨越。健全完善“以工促农、以城带乡”的长效机制，着力推进城乡一体化，大力发展优质高效节水现代特色农业，不断提高农业产业化、规模化、标准化发展水平，持续增加农民收入，加快推进农业现代化进程。加强基础设施建设，力争实现“六个100％、两个80％”的发展目标，农村自来水入户率达到100％，农村社社通油路或水泥路覆盖率达到100％，农村广播电视综合人口覆盖率达到100％，100％的村卫生室达到“三室分设”标准，100％的乡村建成高标准乡镇文化站、村文化室和农家书屋，100％的村建成体育健身活动场所；砖木、砖混结构农宅达到80％以上，末级渠道衬砌率达到80％以上。统筹推进城镇化和新农村建设，全面完成城郊四村改造任务，着力加强永昌县城和朱王堡镇、河西堡镇等小城镇建设，城镇化率提高5个百分点以上，把我市建成国家农村改革市级综合改革试验区。

　　在完善城市功能上实现新跨越。继续推进工业区东扩、生活区北移，加强市政公用设施和城市基础设施建设，加快老城区改造步伐，着力抓好龙首新区和金水新区规划建设，不断增强城市综合服务功能。龙首新区以建设“中央商务区”为目标，重点规划建设商务中心、文化传媒中心、体育中心、生态住宅区和龙首湖生态休闲观光区，努力把龙首新区建设成设计一流、功能完善、设施配套、环境优美、生态宜居的现代化城市新区。金水新区以建设“东部工业港”为目标，重点规划建设企业办公区、企业服务区和商务服务区。

　　在资源环境保护上实现新跨越。大力发展循环经济，推广低碳技术，加快构建资源节约、环境友好的生产方式和消费模式，促进经济社会发展与人口资源环境相协调。积极支持金川集团公司实施资源战略，加大矿产资源开发力度，加强对外合作交流，将资源优势进一步转化为经济优势。加强水资源合理开发、高效利用和有效保护，建设应急备用清洁水源；加强生态环境建设，森林覆盖率提高到22.5％以上，市区绿化覆盖率达到38％以上，力争把我市建设成全国内陆河流域生态文明和节水型社会建设示范区。扎实推进节能减排、污染防治和生态保护，确保二氧化硫、氮氧化物、化学需氧量、氨氮排放量在控制指标以内，万元生产总值能耗年均下降2.3％，城市污水处理率、垃圾无害化处理率分别达到90％和100％。

　　在保障改善民生上实现新跨越。坚持把发展成果体现在提高人民生活水平上，体现在满足人民物质文化需求上，让公共财政的阳光普惠民生，努力促进社会就业更加充分、社会保障体系更加健全、收入分配更加合理、劳动关系更加和谐、公共服务更加便捷。继续抓好教育资源优化整合，注重发展学前教育、特殊教育和职业教育，在全省率先实现“高中向城区集中、初中向城镇集中、小学向乡镇集中、教学点向行政村集中”等“四个集中”目标。加强基层医疗卫生体系建设，促进基本公共卫生服务均等化。加强民主法制建设，促进社会和谐稳定。

　　在推进改革开放上实现新跨越。坚持把改革创新作为推动跨越发展的强大动力，加快推进城乡综合配套改革、医药卫生体制改革、社会管理体制改革、文化体制改革、国有企业改革、事业单位改革、财税体制改革、价格改革等。大力发展文化事业和文化产业，扎实推进国家公共文化服务体系示范区创建工作，着力构建充满活力、富有效率、更加开放、有利于文化科学发展的体制机制，促进文化繁荣发展。加快“人才特区”建设步伐，调整优化人才结构，为经济社会发展提供人才智力保证。健全完善招商引资和争取项目机制，促进非公经济快速发展，引进资金年均增长10％左右。认真落实《金昌—武威区域经济一体化发展规划》，加快建立产业协同发展和重大项目布局、基础设施建设、生态环境共建和公共服务资源共享的体制机制，促进两市共同实现经济社会跨越式发展。

　　三、2012年政府工作的主要任务

　　2012年，是新一届政府履行职责的第一年，也是承前启后、实现“十二五”跨越发展目标的关键一年。政府工作要按照中央、省上的总体部署和市第七次党代会精神，统筹安排，突出重点，努力促进经济社会又好又快发展。

　　主要预期目标是：生产总值增长15％；规模以上工业增加值增长17％；全社会固定资产投资增长20％；大口径财政收入增长12％；地方财政收入增长10％；社会消费品零售总额增长17％；进出口贸易总额增长20％；城镇居民人均可支配收入增长13％；农民人均纯收入增长12.5％；居民消费价格总水平涨幅控制在4％左右；万元GDP能耗下降3％以上；城镇登记失业率控制在4％以内；人口自然增长率控制在7‰以内。围绕上述目标，重点做好以下六个方面的工作：

　　（一）努力抓好项目建设，进一步增强经济社会发展后劲。牢固树立“没有项目就没有发展、没有大项目就没有跨越发展、没有好项目就没有科学发展”的理念，全力以赴加大项目建设力度，千方百计促进经济快速发展。

　　科学谋划项目。立足我市资源禀赋、产业基础和比较优势，认真研究国家宏观政策、产业导向和投资需求，围绕优势传统产业改造提升、战略性新兴产业培育、生产性服务业发展和国内外产业转移承接，突出有色金属精深加工、循环产业链延伸和有色金属粉体材料、盐类化学品、压延加工等新材料，以及风电光电新能源、旅游产业开发、基础设施建设、社会事业发展等方面，科学谋划包装一批环境污染少、经济效益好、带动能力强的重大战略性项目，形成“谋划—包装—申报—实施—储备”的项目梯次推进工作链，着力提高项目对优质资本的吸引力、对优势资源的聚合力，力争谋划储备投资过10亿元的项目10个。

　　全力争取项目。紧紧抓住国家支持甘肃发展、新一轮西部大开发、全国老工业基地调整等政策机遇和国内外产业转移的历史机遇，加大“进京跑部、进省跑厅”项目争取工作力度，重点抓好金阿高等级公路、兰州—张掖客运铁路线金昌段、祁连山水源涵养区综合治理、甘肃电投2&times;660兆瓦热电联产、“引大济西”引水工程等战略项目前期工作。充分利用国际国内两个市场、两种资源，采取会展招商、中介招商、地企联合招商等有效方式，着力在引大引强上下功夫，重点引进镍基合金、电子材料、光电光热装备制造等新材料、新能源和高载能项目，形成“成熟一个、引进一个、带来一批”的集聚效应。

　　精心建设项目。重点实施石羊河流域重点治理、金武高速公路、市区2&times;330兆瓦热电联产、供热管网改造、三峡新能源公司一期10万千瓦风电项目、330千伏河西堡变电站、华能20兆瓦光伏发电、甘肃有色冶金职业技术学院二期、金昌大剧院、金昌博物馆等10个投资过亿元项目。积极支持金川集团公司金川东部贫矿开采、1万吨羰基镍、30万吨铜杆线等项目建设，6万吨电解镍建成投产。开工建设3万吨三氯乙烯、5000吨动力锂电池和100万只圆筒印花镍网等项目，建成车用天然气、中科5兆瓦光伏发电、110千伏西坡变、110千伏水源输变电等项目。

　　严格管理项目。始终坚持“百年大计、质量第一”的方针，健全完善项目招投标、项目法人负责、监理单位控制、施工单位保证、政府监督相结合的项目管理机制，确保每项工程都成为精品工程、阳光工程、廉洁工程，都能够经得起历史的检验和百姓的评判。认真落实市级领导包抓责任制和重大项目例会制，完善重点项目包抓奖惩机制，建立包抓项目定期公示、挂牌督办、项目前期和建设工作责任制度，重点实行市级有10个以上投资过亿元、县（区）有10个以上投资过5000万元、每个综合经济部门有10个以上投资过2000万元、每个社会事业部门有10个以上投资过百万元的“四个十”项目层级推进责任制，建立一个重点项目、一个包抓领导、一套工作班子、一抓到底的项目工作机制，努力形成全市上下齐心协力抓项目、争投资、促发展的良好格局。

　　（二）大力发展循环经济，进一步推动工业经济提质增效。坚持工业立市、工业强市、工业富市，以建立现代工业产业体系为目标，大力发展高端高质高效产业，促进产业转型升级，加快工业由大变强步伐。

　　狠抓工业园区建设。遵循“工业园区化、园区产业化、产业集群化”的发展思路，编制完善金川新材料工业园、河西堡化工循环经济产业园、永昌工业园发展规划，强化园区功能定位，细化区域产业分工，优化特色产业布局，积极培育和引进一批有色冶金新材料产业的大项目以及产业关联度高、带动力强、发展潜力好的大企业，着力培育有色金属、化工、能源、装备制造、建筑材料、冶金、新材料、新能源和农产品深加工等“九大产业集群”，努力争取成为国家有色冶金新材料加工贸易梯度转移承接地和重要的出口加工区。加快园区供电、供汽、污水处理、天然气利用等基础设施建设步伐，增强园区承载能力。建立完善园区管理运行机制，协调做好金昌海关、金昌出入境检验检疫局设立工作，在金昌开发区设立地税、国税、工商、环保、质监、国土管理等分支机构和综合服务中心，实行封闭运行、“一站式”服务，不断提升园区综合服务功能，努力把园区建成管理科学、充满活力、极具吸引力的工业聚集区。

　　加快循环经济发展。坚持把发展循环经济作为调整经济结构、转变发展方式的着力点，建立完善循环型工业、循环型农业、循环型社会三大体系，促进产业在接续中延伸、结构在延伸中调整。切实提高二氧化硫、氯气、焦炉煤气和电石尾气的综合利用水平，重点抓好镍都实业公司1万吨高品质硫酸铜、新川公司20万吨硫酸钾等硫化工项目，金川集团公司40万吨离子膜烧碱二期、30万吨PVC等氯碱化工项目，日升隆公司120万吨兰炭等煤化工产业项目，氟化工产业链延伸项目，建成金化集团公司20万吨合成氨、金泥集团公司40万吨电石、镕凯公司20万吨电石等项目，着力构建有色行业与化工行业以及其他行业间的循环经济产业链。坚持把工业固废利用作为循环经济上水平的主攻方向，加快全国工业固废综合利用示范基地建设，重点支持金川集团公司实施镍冶炼渣资源综合利用、110万吨铜炉渣选矿、尾矿再选和瓮福化工公司50万吨磷石膏、金泥集团公司日产5000吨水泥熟料电石废渣等固废利用项目。认真总结我市循环经济发展经验，承办好全省循环经济发展现场会。

　　加强节能减排工作。严格落实节能减排目标责任制，建立完善节能减排考核评价体系，大力发展低能耗、低污染产业项目，以高新技术、信息技术和先进实用技术改造提升传统产业，重点支持金川集团公司冶炼系统烟气治理、镍都实业公司高盐废水回收、高锍磨浮节能降耗、钴系统提质降耗改造及永昌发电公司脱硝工程、新川化工公司硫酸钾尾气治理、永昌县污水处理厂中水利用、河西堡镇污水处理、农村环境连片综合治理等“九大环境治理和节能减排项目”。强化对重点领域、重点企业和重点污染源预警监管，加强建筑节能和公共机构节能，推进全社会节能，确保完成节能减排目标任务。

　　支持企业发展壮大。坚持把支持企业发展作为政府工作的重要职责，重点在行政审批、项目申报、建设用地、煤电油运、股改上市等方面为企业做好协调服务工作。全力以赴支持金川集团公司推进科技创新，实施以“三个四”为核心的经营发展战略，推进战略引资和整体股改上市；支持八冶建设集团公司开拓市场，扩大对外承包和劳务输出。加快瓮福化工公司、金泥集团公司、金铁集团公司上市步伐。营造公平有序的市场竞争环境，促进民营经济快速健康发展，不断扩大非公经济总量。积极吸引各类金融机构在金昌设立分支机构，抓好中小企业担保中心改制工作，开工建设中小企业公共检测服务中心，筹建市中小企业创新创业服务中心。建立健全市属国有企业负责人收入分配激励约束机制，促进国有企业改革发展和国有资产保值增值。

　　（三）持续推进城乡一体化，进一步提升新农村建设水平。认真落实强农惠农政策，坚持稳粮保供给、增收惠民生、改革促统筹、强基增后劲，促进农业增效、农民增收、农村繁荣。

　　大力发展现代农业。以现代农业示范园区建设引领传统农业改造升级，大力调整产业结构，壮大特色产业，优化产业布局，推进农业现代化进程。着力建设现代农业示范园区。分区域、高标准建设国家级啤酒大麦生产示范区、永昌清河现代农业示范园、金川高效节水农业示范园、金川现代畜牧产业园和城郊“菜篮子”生产基地、沿山冷凉灌区食用菌生产基地，努力使现代农业示范区发展成为现代农业生产的样板区、农业科技成果的展示区、农业经营体制机制创新的试验区和农民接受新知识新技术的培训基地。着力发展优质高效节水现代特色农业。在稳定粮食播种面积、提高单产、增加总产的基础上，重点抓好“千亿斤粮食增产”、节水灌溉、“菜篮子”、畜牧养殖业、设施农业、科技示范等项目建设，推广垄作沟灌38万亩、全膜沟播5万亩、膜下滴灌5000亩；新建改造日光温室、食用菌棚各1000座，塑料大棚5000亩；新建标准化养殖小区20个、万只羊场3个、万头牛场1个、千头牛场2个；培育农业科技示范点25个，示范项目100个，示范面积10万亩。着力推进农业产业化经营，围绕啤酒麦芽、优质肉羊、优质肉牛、优质面粉、无公害蔬菜、食用菌、油料、饲料等产业发展，着力培育一批农业产业化龙头企业、一批专业合作组织和一批农产品营销大户。健全完善农业科技服务体制机制，大力开展下乡科技服务活动，推动农业发展方式转变。着力加强农业标准化生产。建设市级农产品安全检验检测中心和粮食质量检测站，健全完善农产品质量安全监管体系，无公害农产品、绿色食品、有机农产品和农产品地理标志“三品一标”覆盖率达到70％以上。着力提高农业综合机械化水平。改善农机装备结构，农机总动力达到91万千瓦，农业机械化综合作业水平达到78％。着力增加农民收入。进一步加大农业投入，加强农民实用技术培训，提高农民创业就业能力，发展农村二三产业，提高农业组织化程度和农产品市场竞争力，努力增加农民务工、经营和财产性收入。输转城乡富余劳动力8万人次，实现劳务收入8亿元。

　　改善农村基础条件。以石羊河流域重点治理项目为龙头，认真实施灌区续建配套与节水改造、末级渠系配套等水利工程，西金干渠改扩建工程建成投运，新建改建干支渠道100公里。把村庄整治、宅基地置换、富民产业培育和新农村建设结合起来，加快城中村综合改造步伐，新建12个规模适度、功能配套的综合性新农村集中居住区；新建改建农村公路100公里，建设高标准农田1万亩，改造中低产田5000亩。继续打好生态安全保卫战，围绕南部祁连山水源涵养林保护区、中部绿洲农田防护林区和北部戈壁荒漠生态保护区建设，重点实施“三北”防护林、天然林保护、退耕还林、国家重点生态公益林、石羊河流域防沙治沙及生态恢复等生态林业工程，切实保护好水源生命线和北部生态安全屏障。

　　深化农村各项改革。围绕建设国家农村改革市级综合改革试验区，全面推进统筹城乡综合配套改革，深入推进劳动就业和社会保障制度、户籍制度、土地制度、行政管理体制、公共财政体制、规划管理体制等改革，争取三年内实现重点突破、五年内初步形成统筹城乡发展的体制机制新格局。引导农民依法流转土地承包经营权，流转土地达到15万亩以上。按照“政府投资建基础、公开招标引业主、扩大规模增效益”的思路，整合财政支农投入，改革农业投资方式，使支农惠农资金发挥“乘数”放大效应，促进设施农业发展。推进水权交易市场建设，促进水资源向高效益行业配置。改善和创新农村金融服务，鼓励发展龙头企业互助联保、龙头企业+专业合作组织+农户、“心连心”农户联保等金融助农模式，有效解决农村发展资金不足问题。

　　（四）不断强化城市规划建设和管理，进一步提升城市品位。围绕创建国家园林城市、国家健康城市、国家公共文化服务体系示范城市、国家环保模范城市、全国法治城市，进一步巩固和扩大“五城联创”成果，不断完善城市综合服务功能，提升城市形象和品位。

　　高起点规划城市。按照“城市布局科学化、建筑风格特色化、居住环境生态化、综合功能配套化”的要求和“规划先行、适度超前”的原则，通过聘请一流专家和知名机构参与城市规划编制，提高城市规划的完整性、前瞻性、可操作性。编制和完善城市综合交通体系规划、城市综合管网规划、城市绿地系统规划、城市视觉系统规划、城市色彩系统规划等城市专项规划，深化龙首新区、金水新区、老城区等修建性控制性详细规划及城市设计、龙首新区龙形水系景观规划、金水新区绿化景观规划等，突出做好博物馆、文化馆等重点建筑方案的规划设计，做到与城市分区功能定位、资源环境和产业基础相协调。继续做好小城镇和乡村规划编制工作，村庄规划覆盖率达到100％。严格执行规划，完善规划监督管理制度，切实维护规划的连续性、权威性、严肃性。

　　高标准建设城市。围绕龙首新区和金水新区规划，建设金昌文化馆、金昌图书馆、传媒中心、体育中心、老年人康复中心、龙首湖和企业办公楼、酒店、宾馆等，认真做好龙首新区路网和管网工程建设。积极创造条件，采取多种方式，改善机关事业单位职工住房条件。改建行政办公中心，加快上海路沿街、盘旋路周围、14区居住小区等老城区改造步伐，新建南环路等10条城市道路，进一步优化和完善公共设施服务体系。切实落实公交优先发展政策，加强公交站点建设，提高公共交通服务水平。继续实施城市绿化、美化、亮化工程，重点实施西部防护林工程一期配套、龙泉景观带二期、国家级矿山公园等城市绿化工程，加大街景综合改造和夜景亮化力度，不断提升城市品位。

　　高水平管理城市。按照“数字化、网格化、精细化、人性化”的要求，坚持严管重罚和教育疏导相结合，健全完善城市管理长效机制，实行城市管理相对集中处罚权。统筹规划建设交警、城管、国土、治安等信息系统，加快“数字金昌”建设进程。加大城市综合执法力度，组织开展交通秩序、市场秩序、环境卫生、市容市貌等专项整治活动，严格落实“门前四包”责任制和垃圾清扫保洁、日产日清及垃圾无害化处理等制度。探索建立城管部门、社区和相关部门单位有机衔接的网格化管理机制，强化市民文明卫生素质养成教育，实现城市环境美与人文美的协调统一。

　　高效能经营城市。树立“以城养城、以城建城”的现代城市经营理念，按照“政府主导、市场运作”的思路，探索建立城市有形资产转让、拍卖、租赁、置换的有效途径，充分发挥市建设投资开发集团公司的职能作用，积极吸纳社会资金参与城市公用设施建设，盘活城市有形资产，多渠道筹措城市建设资金，重点在盘活金昌毛纺厂、金昌市汽车站等有形资产上取得突破。强化土地储备，加大对城区征用土地、存量国有土地、收回收购土地以及旧城开发整理土地的经营力度，促进城市建设由“政府主体”向“社会主体”转变，由“单一建设”向“整体经营”转变，增强城市资金流、人才流、信息流、物资流的吸引力和凝聚力。

　　（五）积极发展现代服务业，进一步推动第三产业发展上档次。围绕打造金昌城区商贸中心与科技研发中心、永昌城关镇农产品交易中心、开发区汽车服务业集聚区、河西堡镇物流园区、北海子—金水湖城市休闲度假区、圣容寺—骊靬遗址—巴丹吉林沙漠旅游带、龙首新区商业中心和餐饮中心，提升第三产业发展水平，增强对经济增长的拉动作用。

　　改造提升传统服务业。重点实施北京路市场、金三角市场改扩建工程，开工建设大润发超市，建成金川区天庆惠民综合市场。着力发展生活性服务业，扎实推进“万村千乡”市场工程，加快标准化菜市场改造和农村流通网络体系建设，推动商贸服务业提档升级。认真落实“家电下乡”和农机具购置补贴政策，开拓农村消费市场。

　　积极发展现代服务业。以建设“河西走廊区域物流中心”和“西北地区新材料集散中心”为目标，积极发展“通道经济”，重点建设太西煤5000万吨物流园区、镍都实业公司仓储及物流中心和金川区双湾物流中心。加大物流基础设施建设投入，建立完善物流业市场体系和市场管理机制，促进物流业健康发展。积极发展社区服务、养老服务和市政公用事业，加快建设金昌市机动车维修城，大力发展金融保险、电子商务、科技服务、信息服务等产业。

　　加快发展旅游业。按照“政府主推、市场运作、社会参与、部门联动”的工作思路，围绕工业探奇、骊靬探秘、沙漠探险、古迹探幽，着力推进骊靬文化、镍都工业、北海子、御山峡和城市旅游等旅游区建设，实施三角城考古遗址公园项目建设，新建骊靬大道，支持骊靬古城建设，开拍《消失的罗马军团》等影视作品，精心编排《骊靬神韵》大型歌舞剧目，努力扩大骊靬文化知名度。完善旅游基础设施，研究开发特色旅游产品，深化金武阿区域旅游合作，加大旅游资源的策划包装和宣传推介力度，着力打造精品旅游线路。

　　推动文化大发展大繁荣。认真贯彻十七届六中全会精神，统筹城乡文化事业发展，推进广场文化、社区文化、企业文化、村庄文化和校园文化建设，推动文化科技卫生“三下乡”、科教文体法律卫生“四进社区”等活动经常化，促进公共文化服务向农村和基层倾斜。加快重点文化设施项目建设，加强文化市场建设与管理，提高公共文化服务能力。深化文化体制改革，增强文化实力和文化产业发展活力。把社会主义核心价值体系建设融入国民教育和精神文明建设全过程，加强社会公德、职业道德、家庭美德和个人品德建设，为实现跨越式科学发展提供思想道德保障。

　　（六）全力保障和改善民生，进一步加快和谐社会建设步伐。按照“一手抓跨越发展，一手抓保障民生”的要求，进一步加大民生投入力度，不断加强社会管理，促进社会和谐稳定。

　　集中精力办好“十件惠民实事”。 1.新增城镇就业岗位8300个，高校毕业生就业率不低于85％，零就业家庭实现动态消零。2.建立企业职工工资正常增长机制和支付保障机制，城市低保标准提高10％，农村低保标准提高到1400元/年。3.新建廉租房260套、经济适用房612套，城市棚户区改造400套，改造农村危旧房4500套。4.实施农村饮水安全工程，解决 2.1万人的饮水不安全问题。5.积极稳妥地推进公立医院改革，城乡居民医疗保险筹资标准提高到250元/年，新建5所高标准村卫生室。6.完成校安工程10万平方米，新改扩建4所中小学和6所幼儿园。7.改建2个乡镇计生服务所，新建金川区人口和计划生育服务中心。8.新建改造提升乡镇文化站5个、文化室10个、农家书屋15个、乡镇和社区体育健身中心5个。9.建成农超对接便民店10个、社区便民蔬菜店10个、“放心粮油”门店50个。10.完善重要商品价格监测预警机制，适时启动价格调节基金和低保标准与物价挂钩机制，保持市场供应和价格基本稳定。

　　大力实施社会管理创新“六大行动计划”。把社会管理创新摆在更加突出的位置，切实加强社会管理能力建设，下功夫解决离百姓最近、管理难度最大、社会影响最广的突出问题，以均等和普惠实现社会和谐。深入推进平安金昌、法治金昌创建工作，重点实施社会矛盾排查化解、社会治安防控体系建设、食品药品安全、安全生产、预防道路交通事故、城乡环境卫生整洁等“六大行动计划”，有效预防和处置突发事件，推动社会管理规范化、社会化和法制化，努力让全市人民生活得更幸福、更方便、更舒心。进一步密切地企关系，增强服务意识，拓宽合作领域，提高合作水平，巩固发展地企共建共享、互惠互赢的良好局面。加强流动人口计划生育管理和服务，稳定低生育水平。促进妇女儿童、老龄和残疾人事业发展。加强国防、人民防空、民兵预备役建设，深入开展双拥共建，促进军民融合发展。加强和谐寺观教堂建设，依法管理宗教事务，促进民族团结进步。

　　四、加强政府自身建设

　　面对实现跨越式科学发展的新任务，面对人民群众的新期盼，政府全体工作人员惟有敢于负责、全力服务、恪尽职守、清正廉洁，才能不辱使命、不负重托。

　　第一，建设创新政府，增强跨越发展的能力。大力倡导学习之风、调研之风，提倡自觉学习，强化各类培训，不断提高广大公务员特别是各级领导干部的政治理论素养和开拓创新水平，以学立身，以学兴业，争做“学习型城市”、“学习型社会”的精心组织者、积极促进者、带头实践者。继续推进政府职能转变，强化政府社会管理和公共服务职能，善于运用创新的思维、改革的思路、积极的办法，解决好发展中的难点问题和人民群众关注的热点问题，提高推动经济发展、促进社会进步的水平和驾驭全局的能力。

　　第二，建设务实政府，增强服务人民的能力。坚持把务实为民作为政府工作的第一要求，认真落实首问负责制、限时办结制、责任追究制，改进工作考评考核办法，加强对重大决策、重要部署、重点工作的跟踪落实，努力提高政府工作执行力。各级政府领导要带着感情、带着责任，深入基层，深入群众，问政于民、问需于民、问计于民，努力使各项政策措施更加符合发展实际、符合群众利益。坚持一切从实际出发，多做打基础、管长远的工作，多办群众急需、便民利民的好事，说实话、办实事、求实效，努力干出经得起实践、历史和人民检验的实绩。切实精简会议和文件，改进会风文风，严格控制各类庆典活动，使领导干部腾出更多时间抓工作落实，努力提高政府行政效率和服务水平。

　　第三，建设法治政府，增强依法行政的能力。把依法行政贯穿于政府决策、执行、监督的全过程，坚持为民用权、为公用权，严格依照法定权限和程序行使权力、履行职责。自觉接受市人大及其常委会的法律监督、工作监督和市政协的民主监督，加强与各民主党派、工商联、人民团体和社会各界的联系，主动接受社会公众监督，使政府工作人员思想有压力、工作不懈怠，全心全意为发展服务、为基层服务、为人民服务。加强政府信息公开工作和电子政务建设，推进权力公开透明运行。深入开展“六五”普法教育，继续深化行政审批制度改革，确保政府各项工作依法、规范、高效运行。

　　第四，建设廉洁政府，增强防腐拒变的能力。坚持用制度管权、管事、管人，全面落实党风廉政建设责任制，严格执行领导干部问责制、行政执法责任制和经济责任审计制，充分发挥电子监督平台的作用，完善政府投资项目代建制，加强对财政资金、重大投资项目、土地招拍挂、政府采购工作的审计和监察。各级领导干部要牢记廉洁自律的警戒线、党纪国法的高压线，讲党性、重品行、作表率，做到立身不忘做人之本、为政不移公仆之心、用权不谋一己之私。大兴艰苦奋斗之风，努力节约行政成本，控制“三公”支出，把公共财政用在保重点、保运转、保民生上，树立勤政、廉政、优政的良好形象。

　　各位代表，金昌已经迈上新的历史征程，正朝着全面建设小康社会的宏伟目标阔步前进！市政府全体人员要在市委的坚强领导下，在市人大和市政协的监督支持下，凝聚全市人民的智慧和力量，励精图治，奋发图强，真抓实干，为加快建设繁荣文明和谐宜居的现代金昌而努力奋斗！
